# Exploring the impact of intensified multiple session tDCS over the left DLPFC on brain function in MCI: a randomized control trial

**DOI:** 10.1038/s41598-024-51690-8

**Published:** 2024-01-17

**Authors:** P. Šimko, M. Pupíková, M. Gajdoš, P. Klobušiaková, V. Vávra, A. Šimo, I. Rektorová

**Affiliations:** 1 Applied Neuroscience Research Group, Central European Institute of Technology – CEITEC, Masaryk University, Kamenice 5, 625 00 Brno, Czech Republic; 2 First Department of Neurology, Faculty of Medicine and St. Anne’s University Hospital, Brno, Czech Republic; 3 Surgeon General Office of the, Slovak Armed Forces, Ružomberok, Slovak Republic; 4 Faculty of Medicine, Masaryk University, Brno, Czech Republic

**Keywords:** Cognitive ageing, Cognitive neuroscience, Learning and memory, Neural ageing, Visual system

## Abstract

Transcranial direct current stimulation combined with cognitive training (tDCS-cog) represents a promising approach to combat cognitive decline among healthy older adults and patients with mild cognitive impairment (MCI). In this 5-day-long double-blinded randomized trial, we investigated the impact of intensified tDCS-cog protocol involving two trains of stimulation per day on working memory (WM) enhancement in 35 amnestic and multidomain amnestic MCI patients. Specifically, we focused to improve WM tasks relying on top-down attentional control and hypothesized that intensified tDCS would enhance performance of visual object matching task (VOMT) immediately after the stimulation regimen and at a 1-month follow-up. Secondarily, we explored whether the stimulation would augment online visual working memory training. Using fMRI, we aimed to elucidate the neural mechanisms underlying the intervention effects by analyzing BOLD activations during VOMT. Our main finding revealed no superior after-effects of tDCS-cog over the sham on VOMT among individuals with MCI as indicated by insignificant immediate and long-lasting after-effects. Additionally, the tDCS-cog did not enhance online training as predicted. The fMRI analysis revealed brain activity alterations in right insula that may be linked to tDCS-cog intervention. In the study we discuss the insignificant behavioral results in the context of the current evidence in tDCS parameter space and opening the discussion of possible interference between trained cognitive tasks.

## Introduction

Cognitive decline with age and dependency is one of the most challenging problems our society faces in this century. Mild cognitive impairment (MCI) is exceedingly prevalent in the elderly population, with prevalence rates ranging from 16 to 20%^1^. This represents a huge problem because MCI often precedes Alzheimer’s disease (AD)^2^, the leading cause of dementia and cognitive decline in older age^3^ ultimately leading to problems in everyday functioning a introducing a significant burden to both family and caregivers. MCI denotes a neuropsychological construct describing a transitional stage between expected normal cognitive functioning and the early stages of dementia^4,5^, as well as a cluster of diseases that are characterized by impairment in both memory and non-memory cognitive domains with preserved everyday functioning^6^.

Working memory (WM) processes play a vital role in normal aging^7^, MCI, and cognitive decline associated with dementia^8^. While the most common amnestic mild cognitive impairment (aMCI) is primarily characterized by episodic memory disturbances^9^, behavioral and neuropsychological research suggest that aMCI and multidomain aMCI subjects show deficits in working memory^10–15^. WM broadly refers to the cognitive system responsible for the temporary storage and manipulation of information for use in higher-order cognitive processes, such as executive function tasks including planning, reasoning, and decision making^16–18^. WM can also be described as an active and adaptive memory system consisting of multiple components including a module responsible for top-down attentional control, a module for sensory information storage, and a system for updating and manipulating the stored information. It is well established that top-down modulation is a bridging neural mechanism of attention and WM^19^. More specifically, top-down modulation biases the activity of lower perceptual cortices relative to the individual’s goals (e.g., to selectively attend/ignore stimuli like faces vs. scenes). Previous studies employing functional connectivity analysis have demonstrated that the source of top-down modulation in visual working memory involving face and scene stimuli, as well as the underlying neural activity within the visual associative cortices, resides in the prefrontal cortex, encompassing the left dorsolateral prefrontal cortex^20^. Furthermore, top-down modulation is crucial for object identification, especially when objects are presented from non-canonical viewpoints^21^. Previous research has suggested that impaired top-down attentional control of visual information is disrupted in MCI in terms of task-irrelevant information even under low cognitive load^22^. A visual object identification task (VOMT) can estimate the functional integrity of the visual system in neurodegenerative conditions including MCI due to AD^21,23^. Subjects with MCI tend to perform worse in VOMT and their fMRI activations demonstrate over-engagement of specific brain regions and increased functional connectivity as compared to healthy seniors^21^.

Despite concerted global efforts, no treatments (e.g., pharmacological) or strategies to prevent degradation of cognitive functions in MCI are currently available. It is therefore essential to develop alternative strategies, such as cognitive training or bioelectronics, that would boost training gains. The utilization of noninvasive brain stimulation techniques (NIBS), specifically transcranial direct current stimulation (tDCS), has been proposed as a potential non-pharmacological intervention for individuals with MCI, as previous studies have demonstrated its efficacy in enhancing cognitive function and mitigating cognitive decline^24–27^. One limitation of tDCS is that the neuroplastic changes induced by a single tDCS session are typically short-lived, and multiple sessions are often required to produce sustained improvements in cognitive function. Furthermore, the neuroplastic changes induced by tDCS are dose-dependent, non-linear, and complex^28^. Previous studies have suggested that the use of intensified tDCS protocols, such as multiple stimulation sessions per day, increased duration of each session, and alteration of the inter-train period, may enhance the longevity of tDCS effects^29–32^. Engagement in an attention-demanding task manipulates brain plasticity though the mechanism of attentional routing at the time of brain stimulation^33^. The synergy between tDCS with other concurrent cognitive training in age-related neurodegenerative disorders has been investigated and discussed in recent reviews and meta-analyses^24,34,35^. Studies on tDCS-assisted cognitive training (tDCS-cog) have yielded mixed results, ranging from insignificant^36,37^, through positive^38^ to adverse effects^39^. Regarding the stimulation target, the majority of the therapeutic multi-session-controlled trials via NIBS targeted the lDLPFC alone, bi-frontally, or in sequence with other cortical targets^24–26,35^. Previously, we showed that a single session of bi-frontal tDCS with anode targeting the lDLPFC in healthy seniors can positively influence VOMT performance and modulate resting-state brain connectivity^38^. Moreover, an Important consideration in context of aging population is the age-dependent cortical atrophy that increases electrode to brain distance, which gradually reduces electrical field intensity at the target level^40^.

The current study utilizes a multimodal brain stimulation approach that includes multiple sessions of intensified tDCS-assisted visual working memory (VWM) training. Because we investigated the top-down modulation of WM, in our experiment we focused on distinct aspects of VWM: object identity retention and the ability to amplify/suppress task relevant/irrelevant stimuli. The primary objective of this approach is to induce beneficial immediate and long-lasting effects on VOMT in aMCI patients. To gain a more comprehensive understanding of the effect of the intervention, task-induced brain activations were analyzed using functional magnetic resonance imaging (fMRI). As a secondary goal, we investigated whether tDCS can enhance ongoing VWM training in aMCI patients. To the best of our knowledge, no prior study has investigated the efficacy of intensified multiple sessions of tDCS with the aim of influencing cognition in aMCI patients. We hypothesized that the active intensified tDCS group experienced more immediate and long-lasting improvements in VOMT than the sham group. Furthermore, we predicted that the active tDCS group would outperform the sham group during cognitive training. Furthermore, we strived to achieve group homogeneity by conducting a comprehensive neuropsychological assessment and measuring gray matter volumes at baseline, recognizing their pivotal roles in influencing the response to brain stimulation.

## Results

All 35 participants successfully completed the stimulation regimen. No side effects or adverse events were reported by the participants. However, due to excessive head movement leading to compromised data quality, only 34 participants’ fMRI data and 30 participants’ structural magnetic resonance imaging (sMRI) data were included in the analysis. The flow chart of the participants’ follow up can be seen in Supplementary Fig. S3. At the 1-month follow-up, only 28 participants’ fMRI data was available for analysis due to study dropouts. At baseline, we found no significant differences between the groups in terms of age (χ^2^(_32)_ = 129, *p* = 0.44), years of education (χ^2^_(32)_ = 172.5, *p* = 0.55), general cognition as assessed via the Montreal Cognitive Assessment (MoCA) (χ^2^_(32)_ = 119.5, *p* = 0.39), or in any cognitive domain: visual (t_(33)_ = − 0.6, *p* = 0.55), memory (t_(33)_ = − 0.6, *p* = 0.54), attention (t_(33)_ = − 1.69, *p* = 0.1), executive functions (t_(32)_ = − 0.57, *p* = 0.57), language (t_(32)_ = 0.76, *p* = 0.45), and depressive symptoms (t_(31)_ = − 0.053, *p* = 0.95). Furthermore, we found no differences between groups in any of the cognitive tests used in the neuropsychological battery (see Supplementary Table S1).

### Structural MRI results at baseline

Regarding the differences between the experimental groups in gray matter volumes, we found significantly lower volume of the left pallidum in the active stimulation group than in the sham stimulation group (χ^2^_(29)=_192, *p* < 0.01) and a trend toward a smaller volume of the right hippocampus in the active tDCS group than in the sham group (t_(29)_ = 1.93, *p* = 0.06). However, the hippocampus-to-cortex volume ratio (HV:CTV) indicated no differences (t_(29)_ = 0.7, *p* = 0.48) between groups. The rest of the gray matter volumes were comparable between the groups, including the caudate-to-cortical volume (C:CTV) (χ^2^_(29)_ = 99, *p* = 0.42); specific details are presented in Supplementary Table S1.

### Behavioral results

**Figure 1:**
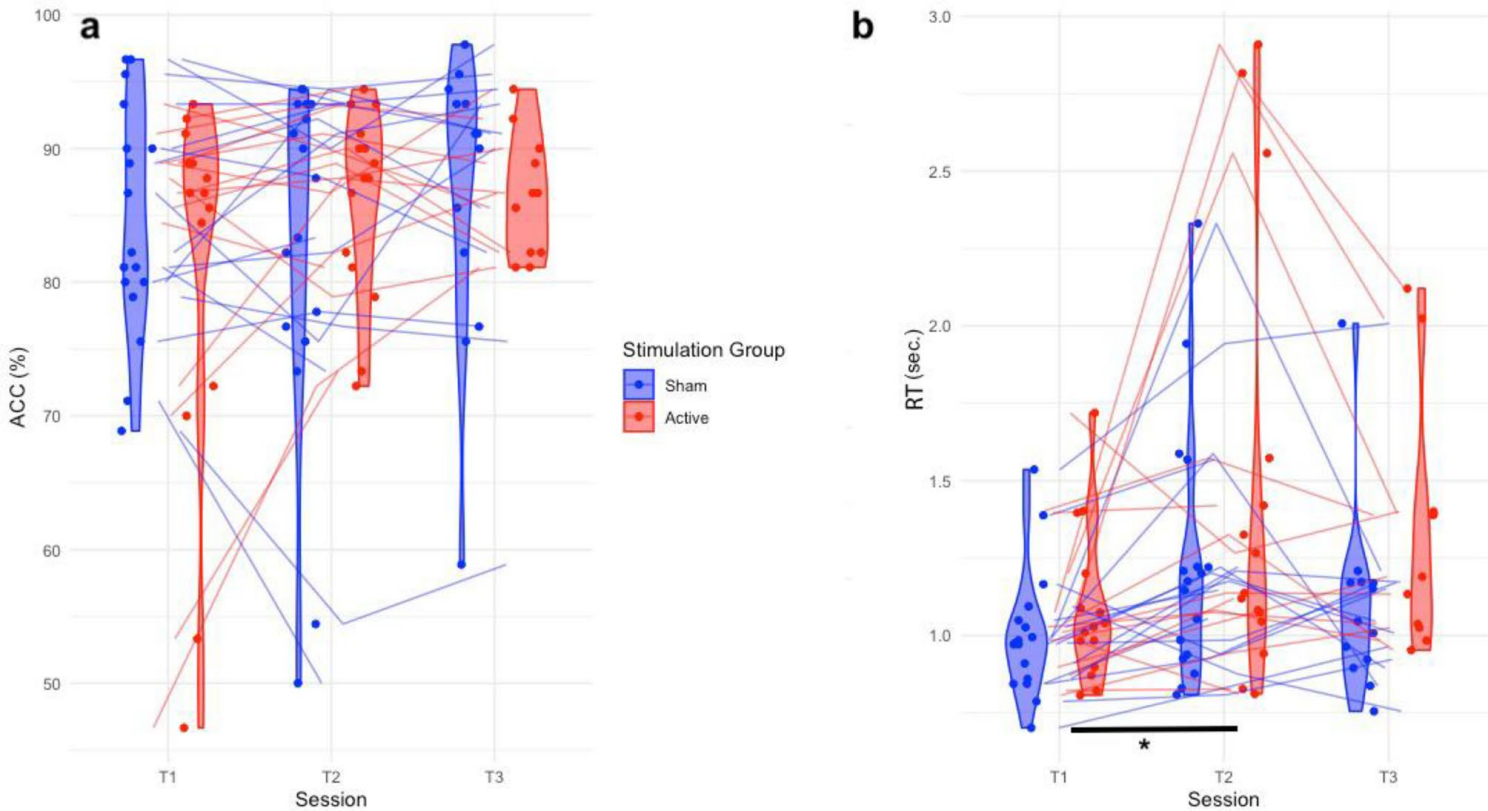
Performance in the VOMT. No significant benefit of tDCS-cog was found in overall ACC and speed (**a**, **b**). Both groups decreased their speed at T1 from T0 (**b**).

Our primary goal was to investigate the effect of intensified tDCS on VOMT changes from baseline to T1 and T2 between experimental groups. Based on the linear mixed-effects model analysis, we found no significant timepoint*stim interaction effect of tDCS on correctness (β = 0.05, SE = 0.09, *p* = 0.59, d = 0.05). Furthermore, both timepoint (β = − 0.02, SE = 0.06, *p* = 0.69) and stim (β = − 0.12, SE = 0.19, *p* = 0.65) factors were insignificant. We performed an analysis to identify intervention responders by calculating Z-scores of changes in mean accuracy (ACC). Our findings revealed that only 5 subjects in the active group and 2 subjects in the sham group exceeded the threshold of 0.3 Z change from T0 to T1. Further details related to responder analysis are depicted in Supplementary Table S3. As for the reaction times (RT), we found timepoint*stim interaction effect (β = 0.06, SE = 0.01, *p* < 0.01, d = 0.06), no effect of stimulation type (β = 0.01, SE = 0.12, *p* = 0.91) and a significant effect of timepoint (β = 0.04, SE = 0.01, *p* < 0.01) demonstrating overall slower responses of both groups as compared to baseline. Specifically, we found a significantly increased RT’s from T0 to T1 in both active (β = − 0.35, SE = 0.02, p_bonferroni_ < 0.01) and sham group (β = − 0.26, SE = 0.02, p_bonferroni_ < 0.01). Lastly, we found a trend toward a weak negative correlation ACC and RT changes from T0 to T1 (r_(31)_ = − 0.277, *p* = 0.06), suggesting a possible trade-off between ACC and RT. Descriptive statistics for VOMT are depicted in Supplementary Table S2 and the plots depicting the results in VOMT are illustrated on Fig.1.

**Figure 2:**
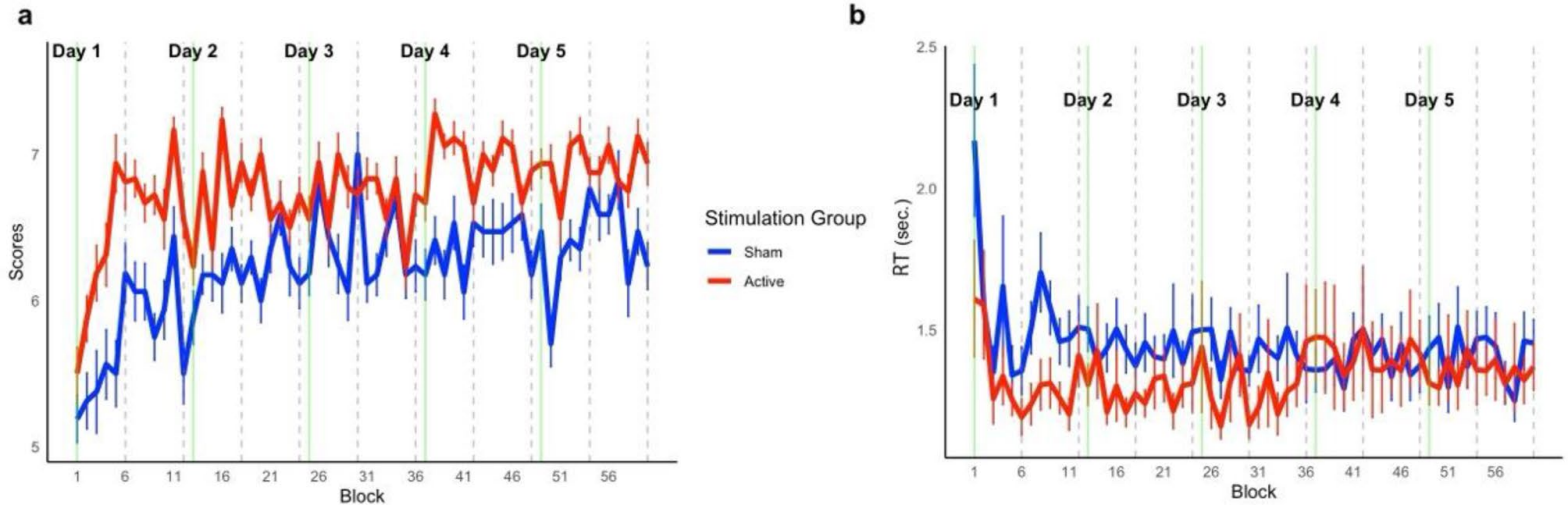
Performance in WMT across all 60 blocks during 5 days of training on V2–V6; (**a**) Changes in outcome score across blocks (Each block consisted of maximum of 8 trials per block) (**b**) Changes in outcome speed across blocks; gray dashed line indicates separate tDCS-cog trains; green line indicates the start of a new training day; Error bars represent standard errors of the mean.

As a secondary outcome, we analyzed the working memory task (WMT) performed concomitantly with active or sham tDCS. We analyzed all datapoints together using LMM and for ACC we found effect of time (β = 0.01, SE = 0.002, *p* < 0.01), but no effect of stim (β = 0.65, SE = 0.39, *p* = 0.11) and no time*stim interaction term (β = − 0.004, SE = 0.003, *p* = 0.14, d < 0.01). As for WMT RT the LMM showed significant effect of time (β = 0.002, SE < 0.001, *p* < 0.01), but no effect of stim (β = − 0.2, SE = 0.11, *p* = 0.08). The time*stim interaction term for WMT RT was significant using LMM (β = 0.003, SE < 0.001, *p* < 0.01, d < 0.01) but this was due high number of comparisons (10 levels for time and 2 for session) as after the necessary Bonferroni correction, we found no significant effects. For additional exploratory results related to WMT we refer to the supplementary materials. To assess the potential transfer effect of tDCS on general cognition, we utilized a LMM and observed no statistically significant time*stimulation interaction term on the Repeatable Battery for the Assessment of Neuropsychological Status (RBANS) (F_(2,62)_ = 0.09, *p* = 0.75) indicating no transfer effects tDCS-cog. Comprehensive descriptive statistics for RBANS across timepoints are provided in Supplementary Table S4 and plotted on Supplementary Fig. S6 (Fig. 2).

### Neural effects and simulation results

**Figure 3:**
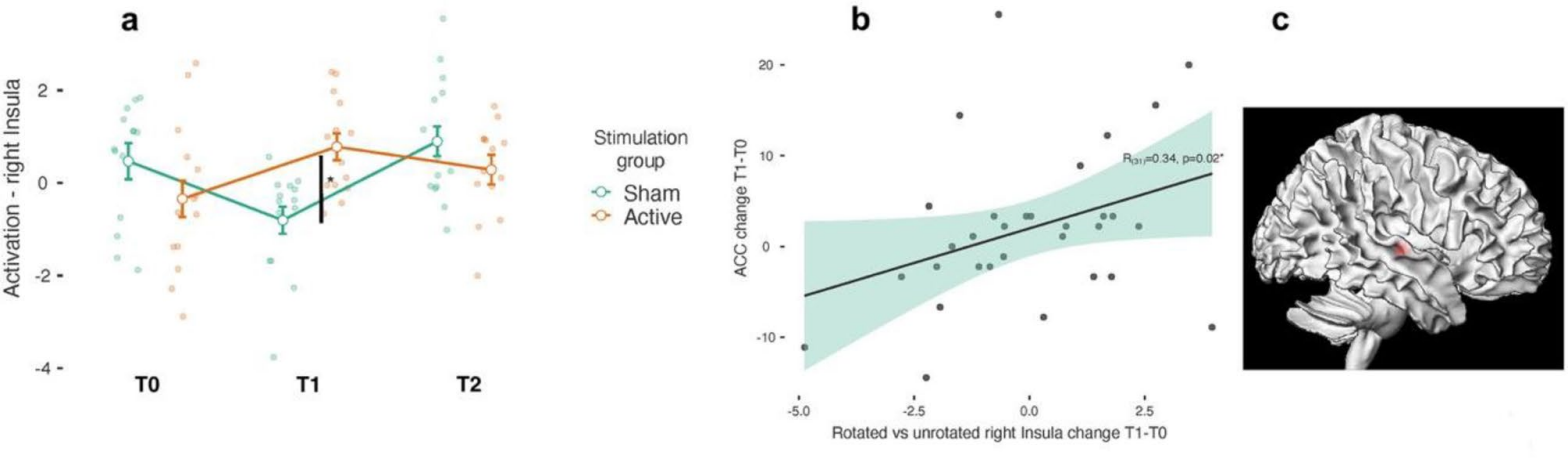
Brain activity changes from Unconventional vs. Conventional view VOMT contrast. The fMRI analysis revealed FWE-corr. cluster of 27 voxels at the right insula. Significantly higher activity in the active group than in the sham group was identified immediately after the end of stimulation period (T1) (**a**). The changes of brain activity correlated with the changes in task performance from T1-T0 (**b**). Location of significant cluster of activation (**c**).

**Figure 4:**
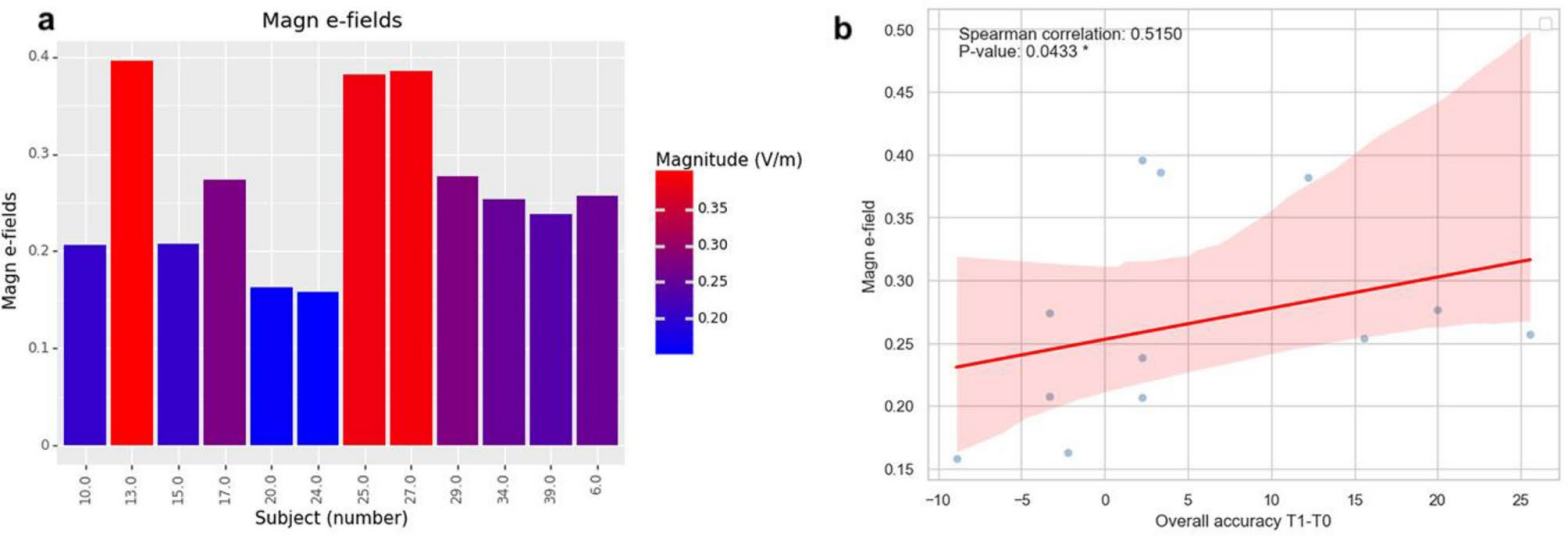
Magnitude of the electric fields across subjects in the tDCS group (**a**). The scatterplot depicts the relationship between tDCS-induced el. fields and VOMT performance change (**b**).

In our fMRI analysis, we investigated the changes in brain activity from relevant VOMT contrast across 3 timepoints and between experimental groups. From the group-level fMRI analysis of cluster-level inference for non-stationary conditions, we identified a statistically significant difference represented by a cluster with maxima at the following coordinates: [30, -16, 1] mm, 27 voxels, p_(FWE-corr)_ = 0.014). Using the NeuroSynth platform^41^, we classified cluster maxima as activation of the right insula. Mixed-design ANOVA revealed a significant time*stim type interaction (F_(2,52)_ = 9,47, *p* < 0.01). A Bonferroni-corrected post hoc test revealed significant higher activity in the active group than in the sham group at T1 (t_(26)_ = − 3.82, *p* = 0.01); there was no such difference at baseline (t_(26)_ = 1,47, *p* = 0.8). Subsequently, we examined the correlation between behavioral changes in VOMT and related brain activity changes. We found a positive correlation between the change in insular activity at T1-T0 and the change in overall accuracy (r_(31)_ = 0.341, *p* = 0.026) in VOMT at corresponding timepoints. The plots depicting then neural effect are illustrated on Fig. 3. Lastly, for the active stimulation group we calculated e-field magnitudes at lDLPFC region of interest (ROI) and found a positive correlation between e-field magnitude and an increase in ACC between T0 and T1 (r_(31)_ = 0.515, *p* = 0.043). The e-field magnitude ranged from 0.158 to 0.396 V/m. The results of el. field simulation are further plotted on Fig. 4.

## Discussion

In patients with amnestic mild cognitive impairment, a 5-day intervention consisting of VWM training with concomitant intensified tDCS with anode placed over the left dorsolateral prefrontal cortex revealed insignificant effect on VOMT performance immediately after stimulation and at the 1-month follow-up and no effect on general cognition as revealed by the results of neuropsychological screening using RBANS. Our data indicate that intensified tDCS-cog with stimulation parameters applied in the current study (2 trains of 2 mA tDCS with 20 min of pause interval) might not influence top-down modulation-dependent visual working memory tasks and does not lead to robust beneficial cognitive after-effects as we predicted. Additionally, the tDCS-cog protocol applied in the current study also failed to induce beneficial effects on online VWM training. The fMRI analysis revealed alterations of task-induced blood oxygen level dependent (BOLD) signal more pronounced in the active stimulation group as compared to sham. Moreover, this change in activation after the tDCS-cog as relative to baseline was related to VOMT task accuracy changes in the active stimulation group. Together, the results of the current randomized trial on patients with MCI revealed no beneficial of tDCS-cog on behavioral performance and might potentially provide some evidence on significant tDCS-cog-induced changes in brain activity during VOMT.

Previous studies that utilized multi-day brain stimulation with cognitive training in both pathological^42,43^ and healthy aging^36,37,44–47^ yielded mixed results. For instance, a recent clinical trial^43^ conducted on MCI-AD continuum patients targeting the lDLPFC demonstrated the enhancement of the cognitive training of face-name associative memory by concurrent rTMS, both post-treatment and at the 2-month follow-up, especially in MCI patients with lower severity of cognitive decline. Compared to our protocol, the mentioned study applied brain stimulation for four weeks; our 1-week intervention was probably not enough to induce long-term effects. Conversely, a relatively small well-designed pilot trial by Das and colleagues^42^ found that tDCS targeted over the left inferior frontal gyrus led to detrimental effects on a 4-week training of executive functions at the immediate and at 3-month follow-up when compared to baseline. The authors also reported increased regional blood flow at the contralateral hemisphere relative to the stimulation area after tDCS as compared to sham.

In the current randomized control trial, we found no support for our main hypothesis that tDCS-cog might induce improvements in VOMT. Contrary to our expectation and in addition to null results in accuracy, we also observed slower reaction times in both groups immediately after intervention. Decreased speed in cognitive tasks among older adults is often reported in the context of speed-accuracy trade-off, resulting from a conscious strategic choice or age-related decrements in brain connectivity^48^. In our data, we found a trend toward a small negative correlation between accuracy and RTs in VOMT that might support an accuracy-speed trade-off; however, this result must be considered with caution as the negative correlation between ACC and speed was small and marginally significant. Alternatively, generally slower RTs may indicate fatigue originating from high demands on the cognitive system by intensive cognitive training during the week. Noteworthily, cognitive demands from joint online and offline cognitive tasks may have interfered with each other, resulting in decrease cognitive speed and no cognitive gains observed across cognitive tasks. Additionally, a correlational analysis between the magnitude of electric fields within the lDLPFC ROI induced by tDCS and VOMT performance revealed a link between accuracy improvements T1-T0 timepoints and the magnitude of e-fields in the ROI in the active stimulation group, implying that the intervention's outcomes may have been determined by el. field strength that was insufficient for inducing observable changes in cognition.

Additionally, tDCS did not augment the VWM training during stimulation. The active stimulation group performed at the level of the sham group although it is crucial to note that during the 1^st^ day of training (we refer to the supplementary materials) the active group improved more than the sham group (specifically between the 1^st^ and 4^th^/5^th^ block of WMT). However, these improvements in WMT likely not reflect tDCS-assisted augmentation of the VWM as we found no time*stimulation interaction for ACC. Interestingly, MCI patients in the tDCS group performed slightly better despite marginally lower cortical thickness of the right hippocampus and left pallidum as compared to the sham group. Potentially, these small group differences in performance in the current trial might originate from better task familiarization in the active tDCS group or other task, training patient-related factors. Importantly, previous studies indicated that patients with more cortical atrophy tend to respond less to brain stimulation^43,49^ and thus this might substantiate why we have not observed no online improvements caused by brain stimulation. We observed a similar pattern for RTs, with a difference of pre-existing baseline differences in between experimental groups.

Regarding the fMRI analysis, immediately following the end of the stimulation regimen we observed BOLD alterations in the right insula, an important node conveying salient information to the DLPFC and responsible for high-level cognitive control via attentional mechanisms and facilitation the access to WM^50,51^. Previously, an ERP study employing a visual-attentional Go/No-go paradigm indicated that early insular activity (pN1 and pP1 components) might reflect attentional and perceptional stimulus processing^52^, cognitive processual components essential for object identification in VOMT^53,54^. In the context of the current research, It must be noted that the observed changes in BOLD activity in the right insula positively linked with changes in VOMT accuracy implying that the observed changes in BOLD activity in the right insula are a neural correlate of tDCS-cog-induced modulation. However due to insignificant behavioral effects in VOMT, it remains a question whether the observed insular activity alteration represents an adaptive neural pattern. Previous studies done in MCI or neurodegenerative dementias reported both increased brain activity reflected as a maladaptive pattern^42,55^ and the neural correlate of task improvement^21,56^. However, these studies employed different brain imaging modalities to estimate brain activity and some of them were also unable to relate the resulting changes in brain activity to behavioral outcomes. While the neural correlates of combined behavioral training and tDCS over the lDLPFC remains to be elucidated, a recent study^57^ adopting both structural and functional neuroimaging showed mounting evidence on tDCS-related alterations in both, white and gray matter microstructure at the stimulated area, as well as functional connectivity alterations within the networks functionally connected to the targeted area.

Contrary to our predictions, we found no long-lasting behavioral or neural effects of repeated intensified tDCS-cog. This might result from significant participant dropouts at the 1-month follow-up resulting in lower statistical power or from not having adequate number of sessions involving tDCS-assisted cognitive training required to elicit long-lasting effects. The expanding research field focused on the tDCS parameter space^29,32^, has offered mounting evidence that higher doses (e.g.: more sessions, stimulation trains, higher stimulation intensity) may not necessarily transfer into better outcomes of brain plasticity and, hence cognitive enhancement. In the present trial, we applied a relatively short 20-min repetition interval between stimulation trains that was previously^32^ shown to more effectively induce a late-phase cortical excitability increase than a longer interval. Several factors differed in the present study, including the application of daily tDCS instead of a single-session protocol, the use of concurrent tDCS-cog instead of tDCS-only, and stimulation over the left DLPFC instead of the motor cortex. While tDCS-cog in MCI subjects has been limited to a few studies^42,58,59^, current evidence in healthy older adults is quite mixed and with exceptions^44,45^, more inclined to the notion that tDCS might not be synergistic with cognitive training^36,37,47,57^. A recent well-designed, pre-registered, and large clinical trial with five days of training provided mounting evidence against tDCS-induced gains in cognitive training involving an decision-making task and untrained transfer task^37^ in healthy older adults. Our current trial extends the knowledge gap in NIBS interventions in MCI subjects by investigating the effects of intensified protocols as opposed to the traditional one train of tDCS per day.

## Conclusions

In summary, the main findings of the current randomized controlled trial indicate that intensified tDCS-cog over the left DLPFC may not result in significant immediate and long-lasting improvements in VOMT in MCI patients with amnestic dysfunction. Our fMRI analysis demonstrated alterations in right insular activity linked to changes in VOMT performance that may be related to tDCS-cog training. The el. current simulation analysis showed significant link between the e-field magnitude in the stimulated ROI and performance change in VOMT. We further found no evidence for the possible tDCS impact on online visual working memory task performance and cognitive transfer effects. The observed slower reaction times immediately after the stimulation regimen points toward a possible interference between online cognitive training and offline VOMT, as utilizing multiple tasks might introduce high cognitive load for MCI subjects.

The current study has several limitations. A larger trial may be required to investigate the true effects of intensified tDCS-cog as the study was done on a relatively small sample size. As a side note, we did not utilize any form of tDCS personalization based spatiotemporal neural readouts to align or interfere with the neurophysiological or pathophysiological processes underlying individual’s distinct physiology, which may have contributed to our inability to induce significant behavioral effects. Another shortcoming of the study might be a potential difference between experimental groups despite random allocation, we discovered differences in terms of their subcortical gray matter volume. However, after adjusting the sMRI outcomes as the ratios to cortical volume, we found no further group differences. Finally, our study does not allow us to parse out the effects of tDCS vs. cognitive training as we did not include a tDCS-only group or sham tDCS with sham cognitive training. The findings of our study warrant further investigation of multimodal approaches with the aim of a deeper understanding of how tDCS titration using intensified protocols influences brain plasticity and cognitive augmentation. In addition to neural effects studied by fMRI, investigation of brain activity dynamics via task-based EEG may shed light on the underlying online mechanisms of electrical neuromodulation.

## Methods

### Study participants

Prior to the study, we determined the required study sample size based on a power analysis leveraging data from our previous research on healthy elderly volunteers^38^, which indicated that a statistical power of 0.95 would necessitate at least 12 subjects per group with a 1:1 ratio. The final sample size of 35 aMCI patients included in the present study corresponds well with previous studies that utilized tDCS or transcranial magnetic stimulation with a similar design^39,60–62^. The study sample consisted of 23 individuals with single-domain aMCI and 12 individuals with multidomain aMCI (aMCI+) with a mean age of 72.4 ± 4.96 years. Patients were recruited from the Neurology Department at Saint Anne’s University Hospital in Brno. Prior to participating in the study, all subjects provided written informed consent and were reimbursed for travel expenses incurred during the final experimental session. The baseline cognitive characteristics are depicted in Supplementary Table S1.

Inclusion criteria for participants in the study were based on the results of a comprehensive neuropsychological examination. The diagnosis of aMCI was established according to Petersen’s criteria^63^, which include: (1) a complaint of memory impairment, preferably corroborated by an informant; (2) impaired memory function (and other cognitive functions in aMCI + subjects) relative to age and education (> 1 standard deviation below the mean) determined as a composite memory domain score derived from multiple memory test; (3) preserved general cognitive function, as determined by scores on the MoCA greater than 24 points and based on the evaluation of a neuropsychologist; (4) intact activities of daily living, as determined by the Functional Activities Questionnaire (FAQ-CZ)^64^; and (5) non-demented status, as determined by the evaluation of a neuropsychologist and a neurologist. Additionally, inclusion criteria were for right-handed individuals between the ages of 60 and 80 years with a minimum of 8 years of education. Participants were also screened for signs of depression using the clinician’s interview and Geriatric Depression Scale^65^ (GDS), and only those without major depressive episode were included in the study.

Exclusion criteria for the study were the presence of ferromagnetic metals in the body, as magnetic resonance imaging (MRI) data acquisition was a component of the study. Furthermore, patients taking medication that could potentially interfere with tDCS stimulation, such as antidepressants, sedatives, anxiolytics, neuroleptics, and other medications, were excluded from the study. Other exclusion criteria were: less than 8 years of education; left-handedness; inability to communicate in Czech or Slovak language; previous neuropsychiatric disorders such as stroke, brain tumor, or cerebral hemorrhage; bipolar disorder, major depressive disorder, schizophrenia, and anxiety disorders; history of substance abuse; previous autoimmune diseases such as multiple sclerosis, fibromyalgia, and rheumatoid arthritis; uncontrolled metabolic disturbances such as uncontrolled diabetes mellitus or thyroid disorders; and other serious diseases that could prevent participation or cause compliance problems, such as cancer patients with a history of radiation or chemotherapy. Demographic data collected included age, sex, and educational level. Participants provided written informed consent in accordance with the ethical guidelines and regulations approved by the ethics committee of Masaryk University.

### Study design

**Figure 5:**
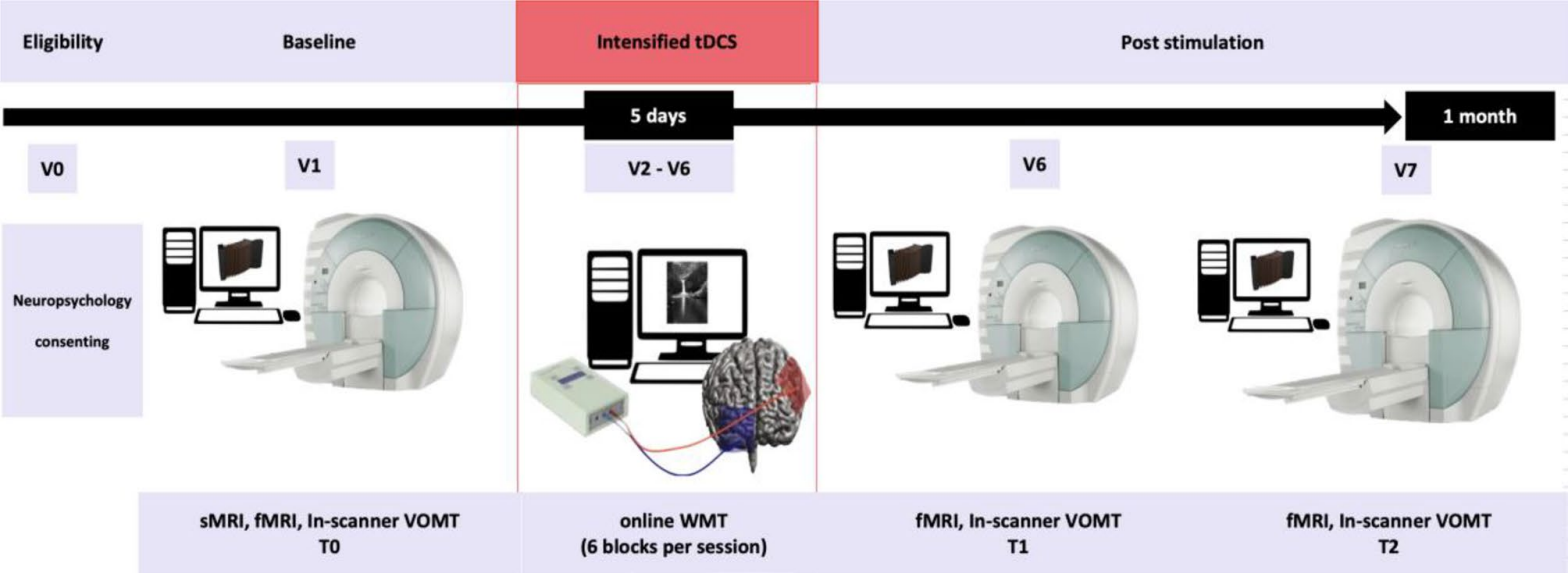
Experimental design. The study involved 7 visits. The active and sham experimental group underwent tDCS-cog (with VWM training) in 5 sessions (V2–V6). VOMT was performed in the MRI scanner prior to and after intensified tDCS-cog and again at 1 month follow-up (V1, V6, V7). V0—baseline, V1—immediately before stimulation, V2–V6—during stimulation, V6—immediately after stimulation, V7—1 month after stimulation.

In this parallel groups randomized controlled trial, the participants were randomly assigned to either an experimental group that received active tDCS stimulation, or a control group that received sham stimulation. Block randomization method was used to generate random allocation sequence with block size at least 12 participants. The allocation sequence was generated by a scientist who was not performing stimulation. The scientist performing tDCS and the MCI patients were blinded to their group allocation. All participants underwent a series of 10 tDCS-cog stimulations delivered over a period of five days, with two stimulation trains per day separated by an inter-train interval of 20 min. Prior to the first stimulation session, participants underwent fMRI data acquisition, which was repeated immediately after the completion of the stimulation series and at a 1-month follow-up. During each fMRI session, participants performed the VOMT inside the scanner as the primary behavioral outcome measure. Additionally, during each tDCS train (together 10 trains, 2 per day), participants performed a WMT with face and scene stimuli as an “online” cognitive training task. Both tasks were practiced by the participants during an “eligibility” session to ensure familiarity with the tasks. The study was approved by the Masaryk University Research Ethics Committee and was conducted in accordance with relevant guidelines^66–68^ and regulations. The trial was pre-registered on ClinicalTrials.gov under the identifier NCT03974087 (04/06/2019). Further details regarding the study are depicted in Fig. 5

.

### Transcranial brain stimulation

Transcranial direct current stimulation was administered using a battery-driven stimulator (DC-Stimulator Plus, NeuroConn GmbH, Germany). The anodal electrode was positioned over the left dorsolateral prefrontal cortex (DLPFC; MNI coordinates: − 40 32 30) and the cathodal electrode was positioned over the right middle frontal gyrus (MFG; MNI coordinates: 44 40 − 10). The precise hotspot of the electrode center in each individual was determined using T1 MRI scan-based frameless stereotactic neuro-navigation targeting with Brainsight™ neuro-navigation software. Despite the relatively large electrode size (rectangular 5 × 5cm), we utilized spatial-navigated targeting because it has been proven in our lab to be less time consuming than traditional targeting methods. An output current of 2 mA was delivered in trains of 20 min per session, with initial and final ramp-up and ramp-down phases of 30 s, respectively. Each participant received 2 trains of tDCS per day, with an inter-train interval of 20 min. The electrodes were secured in place using conductive paste (Ten20 Conductive Paste gel, Weaver and Company) with 1mm thickness. The sham stimulation condition was applied using the same settings; however, the stimulator was turned off after 30 s. Impedance was continuously monitored by the device throughout the session, and stimulation was automatically terminated if impedance exceeded 5 k ohms^69^. The generated e-fields by SimNIBS 4.0^70^ are shown in Supplementary Fig. S1. For the SimNIBS simulation, the ‘charm' command was used to generate the head meshes of all participants using T1 sMRI scans. The current density simulation for each participant in the ’active’ tDCS group was calculated using tDCS parameters: 5 × 5cm electrodes with 5mm electrode thickness and 1mm gel thickness, 2mA current intensity, electrode placements at F3(anode) and FP2 (cathode). We extracted the current densities in V/m from a 1cm^2^ sphere located at the left DLPFC with coordinates corresponding to the targeted DLPFC area.

### Outcome measures

The VOMT served as the primary behavioral outcome measure in our study and has been previously utilized in multiple experimental paradigms to assess VWM function^21,34,38,71^. A visual schematic representation of the task is provided in Supplementary Fig. S2. Different versions of the task were used for every session, with stimuli balanced with respect to difficulty. Accuracy was calculated as the percentage of correct responses. The visual cognitive task comprised unique 60 pairs of randomly presented emotionally neutral images of common objects: 30 with conventional views and 30 with unconventional views (spatially rotated, where one image in each pair was presented in an unconventional rotation, as illustrated in Supplementary Fig. S2, lines 3 and 4). Each trial (together 60) followed this sequence: a 1-s mask stimulus, a 1-s display of the first object, another 1-s mask, a 1-s presentation of the second object, another 1-s mask, and finally, a 5-s fixation cross. Participants observed successive pairs of images. In each pair, the second image could either be identical to the first (conventional condition 1), different in identity (conventional condition 2), the same as the first but spatially rotated (unconventional condition 3), or different in both identity and spatial orientation (unconventional condition 4). Each event type, including conventional conditions 1 and 2, unconventional conditions 3 and 4 occurred fifteen times in the protocol. Using a keypad with 2 buttons, the participants were instructed to respond as fast as they can with focus on responding correctly by pressing a YES button (on the left) if the second object matched the first (regardless of spatial orientation) or a NO button (on the right) if they were different. The conditions were randomly mixed. During the familiarization phase, the participants were instructed to place their right-hand index finger over the left ‘YES’ button and the middle finger over the right ‘NO’ button.

As secondary outcome measure, we utilized a WMT with faces and scenes, (schematic representation in Supplementary Fig. S2) administered concurrently with tDCS (online training task). The WMT consisted of 6 blocks for each train of stimulation, thus participants performed together 60 blocks of cognitive training (2 × 6 blocks per day for 5 days). In each trial, participants viewed sequences of two faces and two natural scenes in a randomized order. The tasks were distinguished by specific instructions that guided participants on how to process the stimuli: (1) "Ignore scenes"—Participants were instructed to remember the faces and disregard the scenes. (2) "Remember scenes"—Conversely, participants were instructed to remember the scenes and disregard the faces. This VWM task necessitated selective attention for encoding the stimuli relevant to the task. After a 9-s delay, participants were evaluated on their capacity to recognize a sample stimulus as one of the task-relevant cues. In total, there were 48 trials, with conditions 1 and 2 randomized across 6 blocks, each comprising 8 trials. Interludes of 5-s pauses occurred within each trial, and 30-s pauses separated the blocks. The entire task lasted approximately twenty minutes, aligning with the duration of a single tDCS train. As stimuli in the task, photographs of faces were obtained from publicly available databases, specifically the Chicago^72^ and Glasgow^73^ face databases. The study participants responded using a keypad with 2 key buttons. Similarly, to VOMT, the participants were instructed to respond as fast as they can with prioritization on the correctness of the response. During the familiarization session, the participants were instructed to place use their right-hand index finger for ‘YES’ response using the left button and middle finger to response ‘NO’ using the right button. The RBANS was employed to assess the potential generalization effect of tDCS-cog on other cognitive domains.

### Data analysis

#### Behavioral data analysis

Statistical analyses were conducted using R with the following libraries, lme4 for LMM fitting, lmerTest for p-values and emmeans for post-hoc comparisons retrieved from CRAN snapshot 2022–01/01. The plots were generated using R package gglplot2^74^. The magnitude of e-fields were calculated using SimNIBS 4.0^70^ and plotted using python Seaborn package used in JetBrains DataSpell Jupiter notebook. As an eligibility criterion, participants were evaluated at baseline using a comprehensive neuropsychological battery. Due to the normal distribution of all variables, we compared the differences between the two groups using independent sample student t-tests across the cognitive domains of visual perception, memory, attention, executive functions, language, and depression. We used a Mann–Whitney test to evaluate any significant differences between the experimental groups in terms of age, years of education, and MoCA scores.

For the primary outcome, VOMT was analyzed using linear mixed models (LMM) with unstructured covariance matrix with timepoint as a fixed effect repeated variable (timepoints: baseline = T0, immediate post-stimulation = T1, 1-month follow-up = T2) and stimulation type (active vs. sham) as a fixed effect factor and subject IDs and trials as random clustering variables using the following formulas: for correctness*—glmer(correctness* ~ stim_type * timepoint + *(1|trial_id)* + *(1|id), family* = *binomial); for RTs—lmer(RT* ~ stim_type * timepoint + *(1|trial_id)* + *(1|id).* LMM were used due to missing data between timepoints. We used ‘glmer’ function for correctness as the outcome for each trial was represented as correct or incorrect. The decision of choosing LMM models including trials as random effects was based on goodness of fit comparison with simple model involving mean ACC/RT, resulting in better fit of the more complex model. In addition, we identified intervention responders in the VOMT by calculating the Z-scores of mean ACC change from T0 to T1 in both groups. More specifically, we identified an individual as a responder if their Z-score of change in ACC exceeded threshold 0.3. The secondary outcome, WMT, was analyzed using LMM with the following formulas: lme(ACC/meanRT ~ time * Stim_type, random = ~ 1|ID, where factor time consisted of 10 tDCS trains. LMM was employed, incorporating session, stimulation type, and their interaction as factors, to examine the potential transfer effect from tDCS-cog to overall cognitive performance. For the analysis of the significant results obtained from the omnibus LMM, the Bonferroni-corrected pairwise comparison of the estimated marginal means was utilized. For LMM interaction terms we calculated the effect size represented as Cohen d’s using a LMMs using formula introduced by Westfall et al.^75^. For exploratory results of both behavioral tasks, we refer to supplementary analysis and results in the supplementary materials.

#### MRI acquisition and preprocessing

Magnetic resonance imaging data were acquired using a 3.0 T Siemens Magnetom Prisma scanner at the Multimodal and Functional Imaging Laboratory (MAFIL) of CEITEC Masaryk University. The fMRI task (VOMT) was acquired using a multiecho multiband T2 echo-planar imaging sequence, with a repetition time of 840 ms, echo times of 14.2, 35.4, and 56.6 ms, voxel size of 2.5 × 2.5 × 2.5 mm, field of view of 180 mm, flip angle of 26 degrees, 60 transverse slices, and 1100 scans, with a multiband factor of 6. Structural MRI was acquired using a T1 MPRAGE sequence, with a repetition time of 2300 ms, echo time of 2.28 ms, voxel size of 0.8 × 0.8 × 0.8 mm, field of view of 256 × 256 mm, flip angle of 8 degrees, and 192 transverse slices. The task-fMRI data were preprocessed and analyzed using SPM12 software running on MATLAB R2019a. The preprocessing pipeline included realignment, multiecho data merging based on contrast to noise ratio^76^, spatial normalization, and spatial smoothing (FWHM 6 mm). The data were checked for spatial abnormalities and artifacts related to excessive head movement using the Mask Explorer tool^77^ and the framewise displacement criterion^78^. Any scans with framewise displacement (FD) greater than 1.5 mm were excluded from the analysis.

#### Structural data analysis

T1-weighted magnetic resonance images were analyzed using the FreeSurfer 6.0^79^ software package (http://surfer.nmr.mgh.harvard.edu). The data were first processed using the “recon-all” pipeline, resulting in segmented images and cortical thickness maps. After each step, the automatic subcortical segmentation labels^80^ and gray-white matter boundaries were visually inspected for each participant. Volumes of segmented subcortical structures, such as the bilateral hippocampus, basal ganglia, and thalamus, were divided by the estimated total intracranial volume (eTIV). The HV:CTV ratio was calculated by summing the left and right hippocampus volumes and dividing by the sum of the left and right lateral frontal cortices (caudal middle frontal gyrus, pars opercularis, pars triangularis, rostral middle frontal gyrus), lateral parietal cortices (superior parietal gyrus, inferior parietal gyrus, supramarginal gyrus), and superior temporal gyri^81^. Finally, the HV:CTV ratio was adjusted using the eTIV. The FreeSurfer cortical structures were defined using the Desikan-Killiany atlas^82^. Similarly, the ratio of C:CTV was calculated as the sum of left and right caudate volumes divided by the sum of left and right lateral frontal cortices (caudal middle frontal gyrus, pars opercularis, pars triangularis, rostral middle frontal gyrus), lateral parietal cortices (superior parietal gyrus, inferior parietal gyrus, supramarginal gyrus), and superior temporal gyri. Both the HV:CTV ratio and C:CTV ratio were then adjusted using the eTIV. The segmentation of cortical structures was performed using the Desikan-Killiany et al. atlas^82^. Structural volumes were then compared between experimental groups using independent sample student’s t-tests or the Mann–Whitney test based on the distribution of the specific variable data. sMRI data were additionally used for modeling the magnitude of e-fields within the targeted lDLPFC using MNI coordinates [− 40 32 30] to define a spherical area with 10 mm diameter.

#### fMRI data analysis

We analyzed the task-fMRI data using the general linear model (GLM) implemented in SPM12. The design matrix in the subject-level analysis included the time courses of task stimulation (observing rotated/non-rotated identical/different objects and control condition) convolved with the canonical hemodynamic response function and head movement parameters (translations and rotations, their differences, squares, and squares of differences) with scans exceeding a FD of 1.5 mm as nuisance regressors. For the following analysis, we prepared differences of the parametric map between visits 2 and 1 (and between visits 3 and 1) in the contrast of observing rotated vs. non-rotated objects. Group-level results were evaluated using cluster-level inference for non-stationary conditions^83,84^ at a family-wise error (FWE) corrected threshold of *p* < 0.05 with an initial cutoff threshold of p (uncorrected) < 0.001.

For subsequent statistical analyses, we extracted GLM activation values for task contrasts on the positions of cluster maxima. The activation value was estimated as average value from a sphere with a radius of 6 mm and the center in a particular position. To understand the alterations in brain activations from the identified cluster maxima across timepoints and experimental groups, we employed a mixed-design ANOVA with time as a three-level repeated within-subject factor (timepoints: baseline = T0, immediate post-stimulation = T1, 1-month follow-up = T2) and stimulation type (active vs. sham) as a between-subject factor. For significant ANOVA results, post hoc tests with Bonferroni correction were applied to determine significant differences between baseline and immediate post-stimulation outcomes, between conditions of active stimulation and sham, and between respective active conditions of stimulation. For the correlation analysis, we first computed the differences between respective timepoints (i.e., T1-T0 and T2-T0) for behavioral measures—VOMT ACC and RT—and the corresponding differences for data activation from the local maxima. Subsequently, we calculated the correlation between changes in VOMT performance and brain activation using the Spearman’s rank correlation coefficient. Lastly, for the correlation between induced magnitude of e-fields and VOMT performance changes, the Spearman’s rank correlation coefficient (one-sided positive) was applied.

### Supplementary Information

Supplementary Information.

## Data Availability

The datasets generated and/or analyzed during the current study are not publicly available in accordance with the informed consent forms signed by the study participants, but they are available from the corresponding author on reasonable request.
