# Supplementary material for: Exploring the impact of intensified multiple session tDCS over the left DLPFC on brain function in MCI: a randomized control trial
Source: Sci Rep. 2024 Jan 17;14:1512. doi: 10.1038/s41598-024-51690-8 (PMC10794210; doi:10.1038/s41598-024-51690-8)
Supplement: Supplementary file 1 — Supplementary Information. [file 41598_2024_51690_MOESM1_ESM.docx]

**Supplementary information**

|  | Domain | | Active | | Sham |  |
| --- | --- | --- | --- | --- | --- | --- |
| N (male/female) |  | | 18 | | 17 | Difference between groups |
| Age (y) |  |  | 72.53±4.13 | | 71.81±5.16 | t_(33)_= -0.722, p=0.47 |
| Education (y) |  |  | 15.05±3.24 | | 14.86±3.61 | 𝜒^2^_(32)_= 172.5, p=0.52 |
| MoCA | General cognition | | 27.5±1.80 | | 27.8±1.48 | 𝜒^2^_(32)_= 119.5, p=0.39 |
| JLO | Visual | | 22.94±4 | | 23.75±4.34 | t_(30)_= 0.556, p=0.582 |
| RO-C | Visual | | 33±3.65 | | 32.43±2.27 | t_(29)_= -0.523, p=0.6 |
| BVMT-I | Memory | | 3.37±1.66 | | 4.33±1.98 | t_(30)_= 1.457, p=0.15 |
| BVMT-D | Memory | | 6.37±2.57 | | 5.43±2.92 | t_(30)_= -0.963, p=0.34 |
| BVMT-R | Memory | | 8.92±4.69 | | 7.93±3.77 | t_(29)_= 0.631, p=0.53 |
| RAVLT-I | Memory | | 36.41±9.05 | | 36.12±9.05 | t_(31)_= -0.095, p=0.95 |
| RAVLT-D | Memory | | 4.68±2.89 | | 5.64±3.1 | t_(31)_= -0.918, p=0.36 |
| RAVLT-R | Memory | | 11.71±4.04 | | 13.9±8.87 | t_(32)_= -1.039, p=0.37 |
| TMT-A (seconds until completion) | Attention | | 46.93±20.74 | | 39.5±14.19 | t_(32)_= 1.223, p=0.23 |
| Digit span, forward | Attention/WM | | 13.27±1.95 | | 13.45±2.06 | t_(20)_= 0.212, p=0.83 |
| Stroop-W | Attention | | 80.55±11.59 | | 77.75±11.22 | t_(32)_= -0.715, p=0.48 |
| Stroop-C | Attention | | 66.55±7.71 | | 63.87±11.46 | t_(32)_= -0.808, p=0.42 |
| Stroop-WC | Executive function | | 33.05±8.71 | | 34.56±14.45 | t_(32)_= 0.373, p=0.72 |
| TMT-B (seconds until completion) | Executive function | | 105±50.88 | | 122.5±42.99 | t_(32)_= 1.076, p=0.29 |
| FPT | Executive function | | 25.88±5.5 | | 26.06±9.45 | t_(32)_= 0.066, p=0.98 |
| VFT-S | Language | | 19.76±9.58 | | 18.5±6.41 | t_(31)_= -0.442, p=0.66 |
| VFT-L | Language | | 38.35±10.7 | | 45.73±10.9 | t_(30)_= 1.930, p=0.06^.^ |
| BNT-30 | Language | | 26.83±4.07 | | 25.33±3.47 | t_(31)_= -1.124, p=0.27 |
| GDS | Depression | | 3.11±2.61 | | 3.06±2.12 | t_(32)_= -0.053, p=0.59 |
| FAQ | Activity of daily living | | 0.5±2.12 | | 0.86±1.4 | t_(32)_= 0.572, p=0.57 |
| Left Hippocampus |  | 0.002±2.868×10-4 | | 0.002±2.073×10-4 | | t_(29)_= -0.722, p=0.47 |
| Right Hippocampus |  | 0.002±1.880×10^-4^ | | 0.003±2.987×10^-4^ | | t_(29)_=1.93, p=0.06^.^ |
| Left Caudate |  | 0.002±4.405×10^-4^ | | 0.002±3.029×10^-4^ | | 𝜒^2^_(29)_= 104, p=0.54 |
| Right Caudate |  | 0.002±3.462×10^-4^ | | 0.002±3.146×10^-4^ | | t_(29)_= -0.68, p=0.53 |
| Left Putamen |  | 0.003±4.051×10^-4^ | | 0.003±4.195×10^-4^ | | 𝜒^2^_(29)_= 126, p=0.89 |
| Right Putamen |  | 0.003±5.201×10^-4^ | | 0.003±3.384×10^-4^ | | t_(29)_= -0.716, p=0.53 |
| Left Pallidum |  | 0.001±5.021×10^-5^ | | 0.001±1.019×10^-4^ | | 𝜒^2^_(29)_=192, p<0.01** |
| Right Pallidum |  | 0.001±9.604×10^-5^ | | 0.001±1.010×10^-4^ | | 𝜒^2^_(29)_= 154, p=0.18 |
| Left Thalamus |  | 0.004±3.018×10^-4^ | | 0.004±5.512×10^-4^ | | 𝜒^2^_(29)_= 123, p=0.92 |
| Right Thalamus |  | 0.004±3.124×10^-4^ | | 0.004±5.154×10^-4^ | | t_(30)_= -0.963, p=0.34 |
| HV:CTV |  | 0.053±0.007 | | 0.055±0.006 | | t_(29)_=0.7, p=0.48 |
| C:CTV |  | 0.051±0.009 | | 0.048±0.006 | | 𝜒^2^_(29)_=99, p=0.42 |

Supplementary Table S1 Baseline characteristics of subjects, cognitive tests within respective cognitive domain, cortical thickness derived from sMRI data, Data provided as means ± SD and range (min to max); Abbreviations: JLO: Judgment of Line Orientation; ROC-C: Rey-Osterrieth Complex Figure Test: copy; BVMT-I, -D; -R: Brief Visual Memory Test -immediate, -delayed, -recall; RAVLT-I, -D, -R: Rey’s Auditory Verbal Learning Test; TMT-A: Trail-Making Test part A; Stroop-W, C, CW: Stroop Color and Word Test, word, color, color-word score; TMT-B: Trail-Making Test part B; VFT-S, L: Verbal Fluency Test, semantic, lexical; FPT: five-point test; BNT-30: Boston Naming Test.

|  | | | | | | | | | | | | | | | | | |
| --- | --- | --- | --- | --- | --- | --- | --- | --- | --- | --- | --- | --- | --- | --- | --- | --- | --- |
| **Experimental group** | | | | **Correct responses** | | | | | | **Speed (sec.)** | | | | | | | |
|  |  | | | **T0** | | **T1** | | | **T2** | | **T0** | | **T1** | | | **T2** | |
| **N** | | Sham | 17 | |  | | 17 |  | 13 |  | | 17 |  | 17 |  | | 13 |
|  | | Active | 15 | |  | | 15 |  | 11 |  | | 15 |  | 15 |  | | 11 |
| **Mean** | | Sham | 76.1 | |  | | 74.3 |  | 77.9 |  | | 1.01 |  | 1.24 |  | | 1.10 |
|  | | Active | 73.0 | |  | | 77.5 |  | 77.8 |  | | 1.09 |  | 1.46 |  | | 1.33 |
| **Median** | | Sham | 74.0 | |  | | 79.0 |  | 82.0 |  | | 0.975 |  | 1.18 |  | | 1.05 |
|  | | Active | 78.0 | |  | | 79.0 |  | 78.0 |  | | 1.03 |  | 1.14 |  | | 1.19 |
| **Standard deviation** | | Sham | 7.74 | |  | | 12.2 |  | 9.78 |  | | 0.208 |  | 0.411 |  | | 0.309 |
|  | | Active | 12.8 | |  | | 6.32 |  | 4.12 |  | | 0.250 |  | 0.707 |  | | 0.402 |
| **Minimum** | | Sham | 62.0 | |  | | 45.0 |  | 53.0 |  | | 0.701 |  | 0.809 |  | | 0.755 |
|  | | Active | 42.0 | |  | | 65.0 |  | 73.0 |  | | 0.807 |  | 0.811 |  | | 0.953 |
| **Maximum** | | Sham | 87.0 | |  | | 85.0 |  | 88.0 |  | | 1.54 |  | 2.33 |  | | 2.01 |
|  | | Active | 84.0 | |  | | 85.0 |  | 85.0 |  | | 1.72 |  | 2.91 |  | | 2.12 |
|  | | | | | | | | | | | | | | | | | |

Supplementary Table S2 VOMT descriptive statistics across experimental groups and timepoints.

| **Subject** | **Stimulation Group** | **ACC change from T0 to T1 (%)** | | **Z-scores of change** | |
| --- | --- | --- | --- | --- | --- |
| 1 | Sham | | 3,33 | | 0,216 |
| 7 | Sham | | 3,33 | | 0,216 |
| 8 | Sham | | -11,11 | | -1,307 |
| 11 | Sham | | 2,22 | | 0,099 |
| 12 | Sham | | -21,11 | | -2,361 |
| 14 | Sham | | -2,22 | | -0,369 |
| 16 | Sham | | -7,78 | | -0,956 |
| 18 | Sham | | -1,12 | | -0,253 |
| **19** | **Sham** | | **14,44** | | **1,387** |
| 21 | Sham | | -6,67 | | -0,839 |
| 22 | Sham | | 1,11 | | -0,018 |
| 26 | Sham | | 3,33 | | 0,216 |
| **28** | **Sham** | | **8,89** | | **0,802** |
| 30 | Sham | | 0 | | -0,135 |
| 35 | Sham | | -14,45 | | -1,659 |
| 36 | Sham | | -2,22 | | -0,369 |
| 37 | Sham | | -3,34 | | -0,488 |
| **6** | **Active** | | **25,55** | | **2,559** |
| 10 | Active | | 2,22 | | 0,099 |
| 13 | Active | | 2,22 | | 0,099 |
| 15 | Active | | -3,33 | | -0,486 |
| 17 | Active | | -3,33 | | -0,486 |
| 20 | Active | | -2,22 | | -0,369 |
| 24 | Active | | -8,89 | | -1,073 |
| **25** | **Active** | | **12,22** | | **1,153** |
| 27 | Active | | 3,33 | | 0,216 |
| **29** | **Active** | | **20** | | **1,974** |
| 31 | Active | | 1,11 | | -0,018 |
| **33** | **Active** | | **4,44** | | **0,333** |
| **34** | **Active** | | **15,56** | | **1,506** |
| 39 | Active | | 2,22 | | 0,099 |
| 40 | Active | | 3,33 | | 0,216 |

Supplementary Table S3 Intervention responder analysis results in VOMT from T0 to T1; Responders marked in Bold

|  | | | | | | | | | | |
| --- | --- | --- | --- | --- | --- | --- | --- | --- | --- | --- |
| **Experimental group** | | | | **RBANS scores** | | | | | |  |
|  |  | | | **T0** | | **T1** | | | **T2** |  |
| **N** | | Sham | 17 | |  | | 17 |  | 14 |  |
|  | | Active | 15 | |  | | 14 |  | 12 |  |
| **Mean** | | Sham | 93.8 | |  | | 94.1 |  | 97.1 |  |
|  | | Active | 92.6 | |  | | 92.92 |  | 94.4 |  |
| **Median** | | Sham | 97 | |  | | 97.8 |  | 99.7 |  |
|  | | Active | 94.5 | |  | | 94.5 |  | 95 |  |
| **Standard deviation** | | Sham | 11.4 | |  | | 13.9 |  | 11.7 |  |
|  | | Active | 9,6 | |  | | 9 |  | 10 |  |
| **Minimum** | | Sham | 72 | |  | | 65 |  | 70.5 |  |
|  | | Active | 74 | |  | | 77.5 |  | 80 |  |
| **Maximum** | | Sham | 113.5 | |  | | 113.5 |  | 121 |  |
|  | | Active | 110 | |  | | 106 |  | 108.5 |  |
|  | | | | | | | | | | |

Supplementary Table S4 Descriptive statistics across experimental groups and timepoints in RBANS

Supplementary analysis and results

In our exploratory analyses, we used mixed-design ANOVA with blocks as a six-level repeated within-subject factor (blocks 1 to 6) and stim type (active vs. sham) as a between-subject factor. The same ANOVA model was applied for both ACC and RT measures of WMT. The model was applied for the data from the 1^st^ session to capture the task performance at baseline and the same model was applied for the remaining stimulation sessions. For all ANOVAs, Mauchly’s test of sphericity was conducted, and where necessary, the Greenhouse-Geisser correction was applied. In case of significant results of the ANOVAs, Bonferroni-corrected post hoc tests were conducted to determine significant differences between baseline and immediate post-stimulation outcomes, between conditions of active stimulation and sham, and between respective active conditions of stimulation.

Based on additional exploratory mixed analysis of variance (ANOVA) of VWM, we found no differences at baseline ACC between the active and sham stimulation groups (F_(1,30)_=1.69, p=0.23) but a significant effect of block (F_(5,110)_=2.88, p=0.01) indicating training effects in both groups during the 1^st^ session. The post hoc test revealed a significant improvement at block 4 (T_(28)_=3.39,p_bonferroni_=0.03) and block 5 (T_(28)_=3.27,p_bonferroni_=0.04) as compared to block 1. We calculated the changes over the blocks for the stimulation sessions 2-10 using mixed ANOVA that indicated an effect of stimulation type favoring the active stimulation group (F_(1,306)_=14, p<0.01, η^2^=0.04) but no effect of block (F_(5,1530)_=0.81, p=0.53) or stim*block interaction effect (F_(5,1530)_=0.74, p=0.59). Regarding the effect of tDCS on **RT**: at baseline, the mixed ANOVA indicated significantly faster responses in the active tDCS group than in the sham group (F_(1,28)_=4.24, p≅0.05). The mixed ANOVA for the remaining sessions revealed a significant stimulation type effect (F_(1,300)_=4.51, p=0.03) favoring the active group but no effect of block (F_(5,1500)_=1.29, p=0.26) or stim*block interaction (F_(5,1500)_=1.82, p=0.1). Plots depicting the results in WMT are illustrated on Figure 3, **Supplementary Fig. S**4 and **S**5.


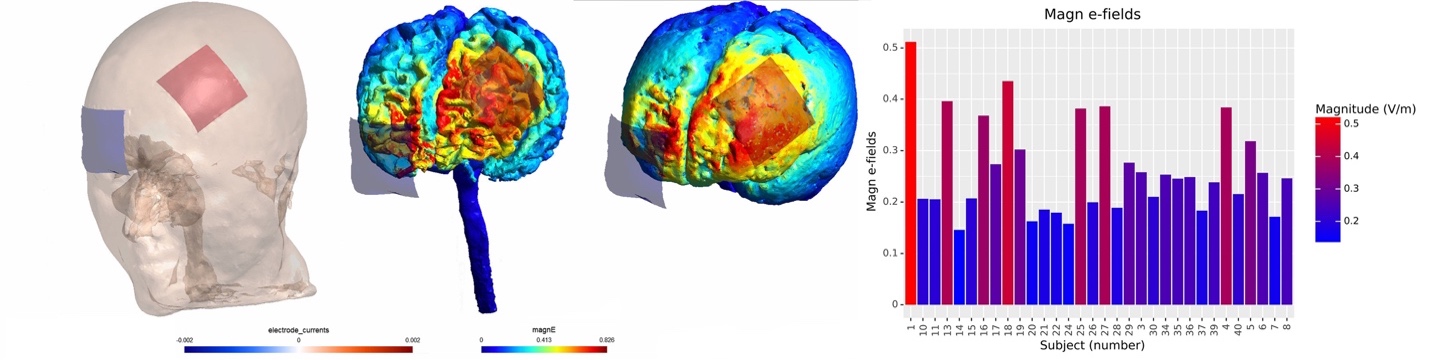


Supplementary Fig. S1 Current simulation of the lDLPFC electrode montage and the simulation of the electric fields on head model. The simulation was built using the male head model and the following stimulation parameters: two square-shape 1 mm thick rubber electrode.


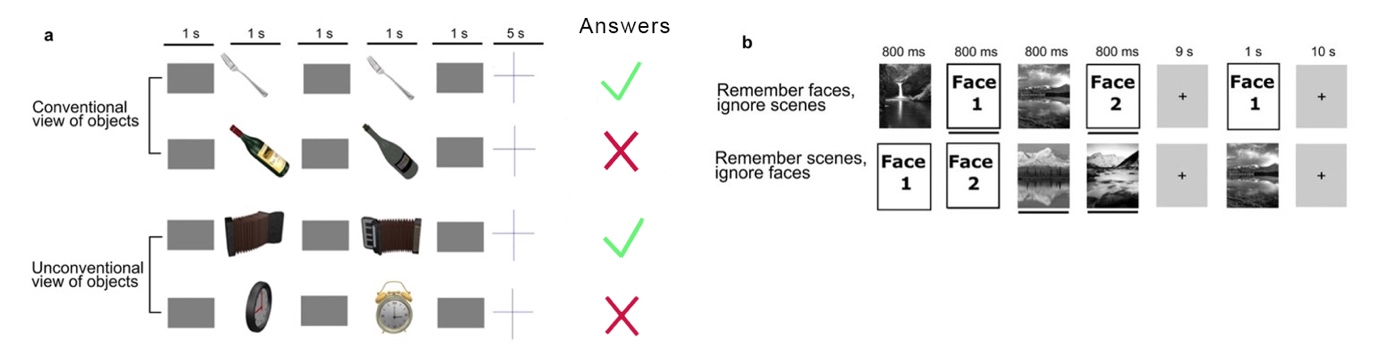


Supplementary Fig. S2 Prior to, immediately after, and at 1-month follow-up after the stimulation, participants performed a VOMT (a). VOMT—subjects respond to whether the two consecutive objects are the same or different by pressing a YES/NO button in two difficulty levels (conventional view of objects—lower difficulty level; unconventional view of objects—higher difficulty level). Online WMT—subjects view a block of faces and scenes (2 + 2, randomized order) preceded by a specific command on how to react to a probe that follows each block. Subjects respond whether the probe is consistent/ inconsistent with the prior instruction by pressing a YES/NO button (b).

**Enrolled N= 35**

**aMCI/aMCI+ patients**

**N=18 (12 aMCI, 6aMCI+)**

Active tDCS

fMRI N=18

VOMT N= 18

sMRI N=17

WMT N=18

**N=17 (11aMCI, 6aMCI+)**

Sham tDCS

fMRI N=16

VOMT N=17

sMRI N=13

WMT N=17

**Active tDCS**

fMRI N=16

VOMT N=18

**tDCS Protocol completion**

**Sham tDCS**

fMRI N=16

VOMT N=17

**1-month follow-up**

**Active tDCS**

fMRI N=14

VOMT N=11

**Sham tDCS**

fMRI N=14

VOMT N=13

**Random group allocation**

**1 fMRI and 5 sMRI subjects excluded** due to movement artifacts during respective sequences

**2 fMRI excluded** due to low data quality

**4 dropouts**

1 Childcare conflict

1 health issues

2 unknown reasons

Additional 3 VOMT dropouts due to scoring by chance

**3 dropouts**

1 Childcare conflict

2 unknown reasons

additional 1 VOMT dropout due to scoring by chance

Supplementary Fig. S3 The flow chart of participant follow-up through the research.


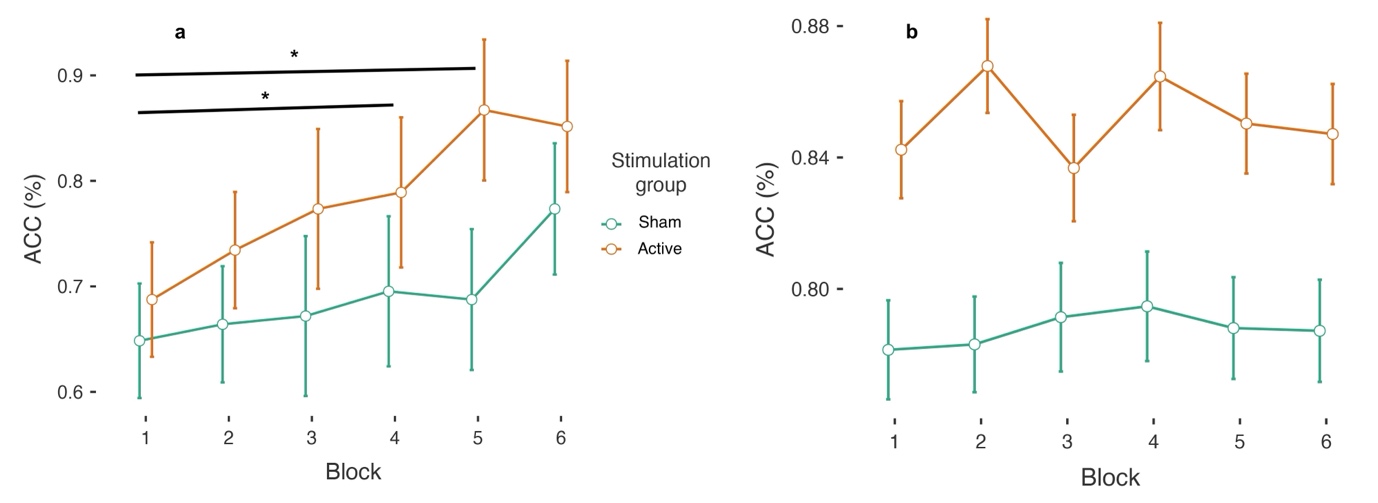


Supplementary Fig. S4 Accuracy in WMT. The block-wise analysis at session 1 revealed no differences between stimulation groups and shows a significant learning in both groups (a). The block-wise analysis from sessions 2-10 showed better ACC of the active tDCS group in performance during all blocks but did not improve over time (b). Error bars represent standard errors of the mean.


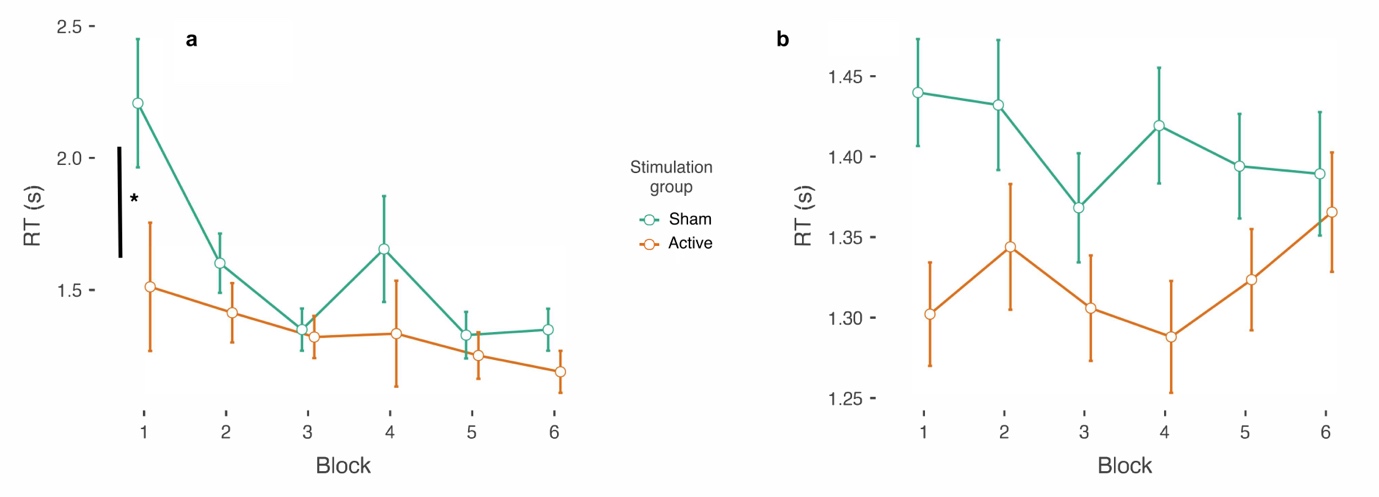


Supplementary Fig. S5 Reaction times in WMT. The block-wise analysis at session 1 revealed significantly higher baseline speed of the active group and gradual improvement of the sham to the level of the active tDCS group (a). From sessions 2-10 the active group responded faster than the sham group but there were no learning gains during the stimulation period (b). Error bars represent standard errors of the mean.


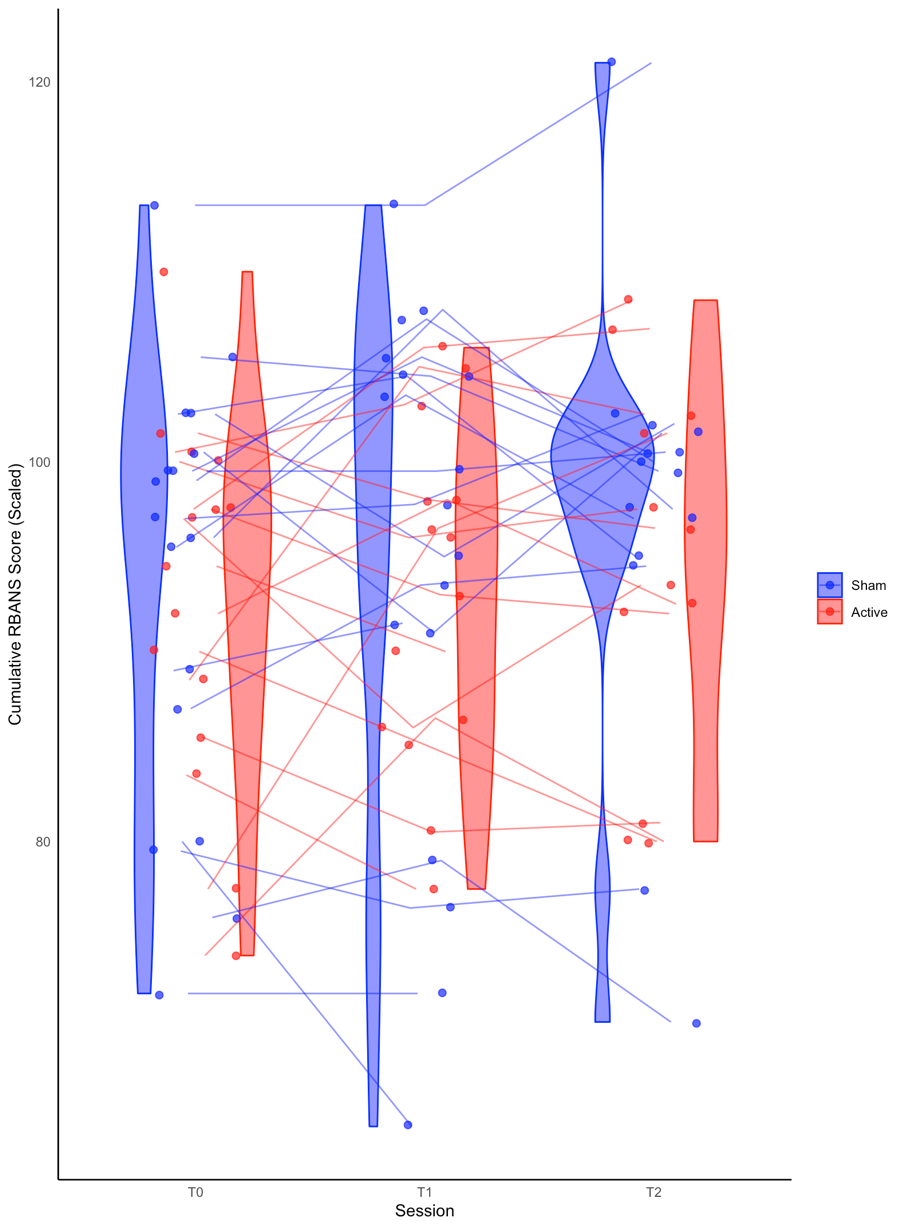


Supplementary Fig. S6 Performance in the RBANS across three timepoints.
